# Symptoms of ADHD in Children with High-Functioning Autism Are Related to Impaired Verbal Working Memory and Verbal Delayed Recall

**DOI:** 10.1371/journal.pone.0064842

**Published:** 2013-05-22

**Authors:** Per Normann Andersen, Kjell Tore Hovik, Erik Winther Skogli, Jens Egeland, Merete Øie

**Affiliations:** 1 Innlandet Hospital Trust, Division Mental Health Care, Lillehammer, Norway; 2 University of Oslo, Institute of Psychology, Oslo, Norway; 3 Vestfold Hospital Trust, Division Mental Health and Addiction, Tønsberg, Norway; University of Texas at Dallas, United States of America

## Abstract

Symptoms similar to those found in Attention-Deficit/Hyperactivity Disorder (ADHD) often occur in children with Autism Spectrum Disorders (ASD). The objective of the current study was to compare verbal working memory, acquisition and delayed recall in children with High-Functioning Autism (HFA) to children with ADHD and typically developing children (TDC). Thirty-eight children with HFA, 79 with ADHD and 50 TDC (age 8–17) were assessed with a letter/number sequencing task and a verbal list-learning task. To investigate the possible influence of attention problems in children with HFA, we divided the HFA group into children with (HFA+) or without (HFA−) “attention problems” according to the Child Behaviour Checklist 6–18. The children with HFA+ displayed significant impairment compared to TDC on all three neurocognitive measures, while the children with HFA− were significantly impaired compared to TDC only on the working memory and acquisition measures. In addition, the HFA+ group scored significantly below the HFA− group and the ADHD group on the verbal working memory and delayed recall measures. The results support the proposition that children with HFA+, HFA−, and ADHD differ not only on a clinical level but also on a neurocognitive level which may have implications for treatment.

## Introduction

The main manifestations of Autism Spectrum Disorders (ASD) (i.e., autistic disorder, Asperger's syndrome and pervasive developmental disorder – not otherwise specified) are impaired social interaction, communication, and restricted and repetitive patterns of behaviours, interests and activities [1]. Although ASD is an exclusion criteria for Attention-Deficit/Hyperactivity Disorder (ADHD) in both DSM-IV-TR [1] and ICD-10 [2] several studies have reported that ADHD symptoms (attention problems and hyperactivity) often occur in subjects with ASD and vice versa [3]–[7]. Comorbidity between ASD and ADHD has been reported to be within the range of 14–78% [4], [5], [8]–[12]. Psychopathological, neurocognitive, brain imaging and genetic studies suggest possible pathophysiological links between ASD and ADHD [13]–[16].

Research has shown different patterns of deficits in executive functioning in children with ASD and ADHD [13]. Children with ASD typically have difficulties with planning and cognitive flexibility, while children with ADHD more commonly struggle with inhibition and sustained attention [13]. However, children with ASD and ADHD-like symptoms seem to display deficits in inhibition similar to that found in children with ADHD [17], [18].

Children with ADHD have been found to perform significantly more impaired than children with ASD on both verbal and visual working memory [19]. In another study only children with ADHD, and not children with ASD, displayed impairments in visual working memory when compared to typically developing children (TDC) [20].Other studies have found no significant differences between children with ASD, ADHD and TDC on visual working memory [21].

Most studies comparing neurocognitive functioning in children with ASD and ADHD have examined performance on working memory and executive functions tasks. To our knowledge, no studies have investigated acquisition (learning) and delayed recall in children with ASD compared to children with ADHD. A review of memory functions in ASD found that memory deficits were related to retrieval problems and not to acquisition problems [22]. In contrast to this, free delayed recall, cued memory and recognition have been found to be intact in ASD [23]. In ADHD research some studies have found more impairments in acquisition than in delayed recall in children with ADHD [24]–[26], while others have found impairments in delayed recall but not in acquisition [27], [28]. Andersen et al. [24] speculated that memory deficits in ADHD may be secondary to attention deficits and reduced effort, causing incomplete acquisition and subsequently impaired delayed recall. Whether this is the case also in ASD is not clear. It is possible that memory impairments are mediated by other mechanisms in ASD, causing recall problems instead of impaired acquisition [22].

Group comparisons can be misleading when heterogeneity is large. A deficit in a subgroup of patients may account for diagnostic group differences. This may be one explanation for the inconsistent findings, depending on the composition of the HFA sample [16]. A recent review stated that more research on possible similarities and differences in neurocognitive functioning between children with pure ASD, children with pure ADHD and children with ASD and clinically significant ADHD symptoms are warranted [13].

The main aim of the present study was to examine verbal working memory, verbal acquisition and delayed recall in children with high- functioning autism (HFA; subjects with ASD with average or above average intellectual abilities) compared to children with ADHD. A group of TDC was also included. The children in the study were between 8–17 years. We wanted to investigate whether the inconclusive findings of previous research could be overcome by dividing the group of children with HFA into subgroups with or without attention problems. Thus, we first tested the overall group difference between the diagnostic groups, expecting to find a deficit in working memory in children with ADHD, but not in children with HFA. On the other hand, we expected to find a deficit in delayed memory in children with HFA. However, by dividing the HFA groups we expected to find that a sizable subgroup of children with HFA and comorbid attention problems (hereafter referred to as HFA+) will have deficits in working memory comparable to the children with ADHD. Further, we expected that memory impairments may be differentially mediated in the sense that the children with HFA+ will show the same pattern of impaired acquisition as children with ADHD, whereas those with HFA without attention problems (hereafter referred to as HFA-) will show a post-encoding deficit in delayed recall.

## Methods

### Ethics statement

Both children (12 years and older) and parents gave informed written consent prior to inclusion. The study was conducted in accordance with the Helsinki Declaration of the World Medical Association Assembly. It was approved by the Regional Committee for Medical Research Ethics in Eastern Norway (REK-Øst), and by the Privacy protection ombudsman for research at Innlandet Hospital Trust.

### Participants

Demographic and clinical characteristics are presented in Table 1.

**Table 1:** Characteristics of the HFA, ADHD and TCD groups with means and standard deviations.

Variable	HFA	ADHD	TDC	Group comparison		Post-hoc ^c)^
	(*n* = 38)	(*n* = 79)	(*n* = 50)	Chi- square/*F*	*p*	
Sex (male/female)	31/7	44/35	32/18	7.5	.024	
Age	12.0 (2.3)	11.6 (2.0)	11.6 (2.0)	1.1	NS.	
Mother's education	12.8 (2.6)	12.7 (2.1)	14.6 (2.4)	11.4	<.001	ADHD,HFA<TDC
CBCL school ^a)^	35.1 (1.1)	33.9 (0.8)	50.2 (1.0)	89.2	<.001	ADHD,HFA<TDC
CBCL total problem ^b)^	63.9 (1.4).	61.8 (1.0)	37.9 (1.2)	139.3	<.001	HFA,ADHD>TDC
VIQ	95.3 (16.4)	93.2 (14.2)	99.3 (12.0)	2.9	NS.	
PIQ	99.6 (17.9)	98.7 (15.4)	107.4 (14.0)	5.1	.007	ADHD<TDC
FSIQ	98.2 (17.8)	95.6 (14.0)	103.8 (13.0)	4.8	.010	ADHD<TDC

*Note*. HFA; High-functioning Autism: ADHD; Attention-Deficit/Hyperactivity Disorder: TDC; typically developing children: VIQ; verbal IQ: PIQ; performance IQ; FSIQ; full scale IQ. IQ estimated measures from the Wechsler Abbreviated Scale of Intelligence (WASI) ^a)^ T-scores; higher score  =  better performance: ^b)^ T-scores; higher scores  =  more problems. ^c)^ Fishers LSD.

The children with HFA and ADHD were recruited from the Child and Adolescent Mental Health Centres in Innlandet Hospital Trust (IHT) in Norway. Diagnostic assessments were based on interviews of participants and parents using the Schedule for Affective Disorders and Schizophrenia for School Age Children/Present and Lifetime version- 2009 (K-SADS-PL) [29].The interviewers were experienced psychologists and educational therapists. Results from the K-SADS-PL interviews and supporting information were reviewed independently by the supervising senior clinician who is a specialised psychologist in neurodevelopmental disorders (M.Ø.). Disagreements were discussed in meetings with all the clinicians (13) present to arrive at a ‘best estimate’ DSM-IV consensus diagnosis. The diagnostic evaluations were supplemented with information from the ADHD Rating Scale IV [30], the Autism Spectrum Screening Questionnaire (ASSQ) [31] and the Child Behaviour Checklist (CBCL) ADHD scale [32], all filled out by the parents. Normative data from the ASSQ [31], from the ADHD Rating Scale IV manual [30], and T-scores above 65 on the syndrome and DSM-oriented scales in CBCL [32] were applied to assess clinical significance. Supplemental information from teachers about the child's school functioning is mandatory on all referrals and was included in the overall clinical assessment. If both parents could not report on K-SADS-PL and rating scales together, information from mothers was used. In cases of disagreement between parents, information from mothers was emphasized. When information on the K-SADS-PL was not consistent with rating scales, information from K-SADS-PL was emphasized in the assessment. All diagnoses had to fulfil the criteria in the DSM-IV [1].

Thirty-eight children and adolescents (31 males, mean age 12.0, range 9–17) with HFA were included. Thirty-one were diagnosed with Asperger's syndrome and seven with pervasive developmental disorder – not otherwise specified. One of the children used a small dose of antipsychotics (aripiprazol 5 mg) due to aggressive behaviour. One child was medicated with stimulants (methylphenidate dosage of 30 mg–2.4 mg/kg). Two participants used stimulant (methylphenidate) medication, but medication was discontinued 24 hours before assessment. Children medicated with stimulants had severe ADHD-like symptoms. None of the participants met the diagnostic criteria for Autistic Disorder. This was probably due to a national referral policy canalising subjects suspected of Autistic Disorder elsewhere. The ADHD group consisted of 79 children and adolescents (44 males, mean age 11.6, range 8 to 16). Forty-four were diagnosed with ADHD-inattentive subtype and 35 with ADHD-combined type. The ADHD diagnostic subgroups were treated as one group in the analyses because there were no significant differences between the subgroups on the neurocognitive measures. Except for two, all of the ADHD children were medication naïve. In contrast to the medicated children in the HFA group, the ADHD children were newly referred at the time of inclusion. Of the two medicated participants in the ADHD group one was medicated with risperidone (0.5 mg), and one with a small dose of quetiapine (100 mg) due to aggression.

The TDC group consisted of 50 children and adolescents (32 males, mean age 11.6, range 8 to 17). They were recruited from local schools and attended regular school classes at normal grade levels. An information letter describing the research project was distributed to all students and their parents by their teachers. Separate K-SADS-PL interviews with children/adolescents and parents revealed no mental disorders among the TDC. The TDC were given a small compensation (a gift-card of approximately 47 US dollars) for their participation. There were no significant differences between the groups with regard to age. There were significantly (*p* = .024) fewer girls in the HFA group compared to the two other groups. The ratio of males to females is similar to that found in prevalence studies [33], [34].

The CBCL [32] yields different measures of emotional and behavioural disturbances in children and adolescents. The school scale and the total problems scale from the CBCL [32] were used to compare school and global functioning between groups. Both clinical groups performed impaired compared to TDC on both measures (*F* = 89.2, *p* <.001 and *F* = 139.3, *p*<.001), and there were no differences between the ADHD and the HFA groups.

The Wechsler Abbreviated Scale of Intelligence (WASI) [35] was administered to estimate verbal IQ (VIQ), performance IQ (PIQ) and full-scale IQ (FSIQ). There was a significant difference between the groups with regard to PIQ (*F* = 5.1, *p* = .007) and FSIQ (*F* = 4.8, *p* = .010). Post-hoc analysis showed that the ADHD group had significantly lower PIQ and FSIQ than the TDC group. The HFA group did not differ significantly from the TDC group or from the ADHD group with regard to the IQ measures. Mothers of TDC had significantly longer education than the clinical groups. However, the education level of the mothers of TDC was nearly equal to that of mothers of TDC in comparable studies in Norway [36].

The HFA group was divided into two subgroups (see Table 2), with (HFA+) or without (HFA-) clinically relevant attention problems based on scores on the CBCL attention subscale [32]. The American clinical borderline cut-point score (T = 65) was applied [32]. In the HFA+ group there were 16 children (13 males) and in the HFA- group there were 22 children (18 males). The HFA+ group had a significantly more impaired score compared to the HFA- group on the CBCL attention subscale (*F* = 5.5, *p* = .025). The HFA+ and HFA- group did not differ significantly with regard to IQ, CBCL composite score (the mean of T-scores from the CBCL syndrome scales), sex distribution or age. The CBCL measure of attention was moderately (*r* = .33, *p*<.05) correlated to ASD symptoms on the ASSQ.

**Table 2:** Characteristics of the HFA + and HFA – groups with means and standard deviations.

Variable	HFA + ^a)^	HFA − ^b)^	Group comparison	
	(*n* = 16)	(*n* = 22)	Chi- square/*F*	*p*
Sex (male/female)	13/3	18/4	.002	NS.
Age	12.2 (2.0)	11.9 (2.5)	3.4	NS.
CBCL attention problems subscale	71.6 (7.3)	57.4 (4.5)	5.5	.025
CBCL syndrome composite scale ^c)^	65.9 (5.4)	61.3 (7.6)	2.5	NS.
FSIQ (*M/SD*)	91.7 (16.4)	102.9 (17.6)	.15	NS.

*Note*. HFA; High-functioning autism: ^a)^ CBCL attention problems t-score >65, ^b)^ CBCL attention problems t-score≤65: ^c)^ CBCL mean syndrome scales scores without attention problems: FSIQ; full scale intelligence. IQ estimated measures from the Wechsler Abbreviated Scale of Intelligence (WASI).

Exclusion criteria for all groups were prematurity (<36 weeks), IQ estimate below 70, and neurological disease. For the ADHD group an additional exclusion criterion was previous stimulant treatment. For the TDC additional criteria were no history of a psychiatric disorder, dyslexia, nor head injury (with loss of consciousness).

### Measures

The Letter-Number Sequencing test (LNS) from the Wechsler Intelligence Scales for Children – IV (WISC-IV) was used to measure verbal working memory [37]. It is considered to be a working memory test involving verbal attention span, mental manipulation, short-term memory, sequencing, visuospatial imaging and processing speed [37]–[40]. The LNS consists of ten items. Each item contains three trials with the same number of digits and letters. Children are required to listen to a presentation of alternating letter and digits. After each trial the child is asked to recall the numbers in ascending order and the letters in alphabetical order [37]. In the current study total correct recalled trials were examined. Lower raw scores indicate difficulties with the task. Factor loading (.62) and test–retest reliability (.70–.80) are reported as good for the LNS [41].

The Norwegian version of the Hopkins Verbal Learning Test - Revised (HVLT-R) [42] was used to assess acquisition and delayed recall. The HVLT-R is a list learning test, which consists of 12 nouns within three semantic groups. The acquisition variable consists of three acquisition trials in which the administrator reads the words aloud and then asks the child to repeat as many as he/she can remember in any order. A delayed recall trial is introduced after 20–25 minutes, in which the child is asked to simply retrieve as many of the words listed in the acquisition trial as he/she can remember. The children are not informed about the delayed recall trial beforehand. Lower raw scores indicate difficulties with the task. Research using HVLT-R has shown good discriminant validity (canonical correlations up to.83) and test-retest reliability ranging from.47 to.59 [43]–[45].

The parents were asked to fill out the CBCL [32]. The CBCL is a 120 item instrument that provides information on several subscales regarding child/adolescent psychopathology and behavioural disturbances. It is widely used internationally and has demonstrated good discriminant validity with mean factor loadings across societies at.62 [46]. Parents rate the items on a three point Likert-type scale with the values: 0 = not true, 1 = somewhat or sometimes true, 2 = very often true. In addition to total problems, school and attention subscales, a composite score (i.e. mean of T-scores from the CBCL syndrome scales without the attention problems subscale) was computed.

### Data Analyses

Significant results are reported at *p*≤.05 level. Demographic characteristics were investigated using the Chi-squared test for independence (gender) and one-way analysis of variance (ANOVA) (age, mother's education, IQ) followed up by Fisher's Least Significant Difference (LSD) post-hoc test for group comparisons. Pearson correlations between verbal working memory, acquisition and delayed recall were conducted for all groups. Differences between the HFA+ and HFA- groups on age, IQ and CBCL syndrome subscales were analyzed with an independent samples T-test. Differences between groups on verbal working memory, acquisition and delayed recall measures were analyzed with ANOVAs followed by Fisher's LSD post-hoc test. To investigate potential differences between the HFA+, HFA-, ADHD and TDC groups, ANOVAs were followed by Fisher's LSD post-hoc test for the verbal working memory, acquisition and delayed recall measures. Separate ANOVAs excluding the children that were on medication were also performed. To control for a possible confounding effect of gender an overall MANCOVA of all dependent measures with sex as a covariate was conducted. We also ran independent measures T-tests between those in the ADHD group with a T-score above 65 on the CBCL attention subscale and those with a T-score below 65. The data analyses were conducted using the Statistical Package for Social Sciences (SPSS) version 19.0 for Windows.

## Results

### The results for the undivided HFA-group, the ADHD group and the TDC group (see Table 3)

**Table 3:** Results on Letter Number Sequencing task (LNS) and Hopkins Verbal Learning Test—Revised (HVLT-R): means and standard deviations within the HFA, ADHD and TCD groups, and results from ANOVAs with post-hoc group comparisons reported, and MANCOVA controlled for IQ.

Variable	HFA	ADHD	TDC	Group comparison			Post-hoc ^c)^	Controlling for IQ ^d)^
	(*n* = 38)	(*n* = 79)	(*n* = 50)	*F*	*p*	η^2^		
LNS	15.0(3.4)	15.5 (3.3)	18.2 (1.9)	*F*(2,164) = 17.0	*p*<.001	.171	HFA,ADHD<TDC	HFA,ADHD<TDC
HVLT-R acquisition ^a)^	21.4 (6.5)	21.1 (5.9)	26,3 (4,7)	*F*(2,164) = 14.1	*p*<.001	.147	HFA,ADHD<TDC	HFA,ADHD<TDC
HVLT-R recall ^b)^	7.5 (2.4)	7.5 (2.3)	9,1 (2,1)	*F*(2,164) = 8.7	*p*<.001	.097	HFA,ADHD<TDC	HFA,ADHD<TDC

*Note*. HFA; High-functioning autism: ADHD; Attention- Deficit/Hyperactivity Disorder: TDC; typically developing children, ^a)^ sum trial 1, 2, 3, ^b)^ recall after 20 minutes, ^c)^ Fishers LSD. ^d)^ *p*<.05 in pairwise MANCOVA controlling for the effect of IQ.

ANOVA revealed significant group effects for all three test scores, i.e. verbal working memory (LNS), (*F* (2,164) = 17.0, *p*<.001); HVLT-R acquisition (*F* (2,164) = 14.1, *p*<.001) and HVLT-R delayed recall (*F* (2,164) = 8.7, *p*<.001). Fisher's LSD post hoc analysis showed that both the HFA group and the ADHD group scored significantly (*p*<.001) impaired compared to the TDC. There were no significant differences between the HFA and ADHD groups. Correlations between working memory, acquisition and delayed recall were large (r≥.50, *p*<.001) in the TDC group and medium within the HFA group (r≥.30, *p*<.001). Within the ADHD group all three measures were also significantly related (*p*<.05) with small to medium (*r*≥.27) correlation coefficients.

The children in the ADHD group scored significantly (*p* = .002) below the TDC on FSIQ. When controlling for FSIQ in pair-wise MANCOVAs, FSIQ was significantly related to all measures, but all group differences from the ANOVAs still remained significant. The HFA and ADHD groups did not differ significantly from each other after controlling for FSIQ. When comparing the HFA and TDC groups, the main effect of diagnosis when controlling for FSIQ were as follows: verbal working memory (LNS) (*F* (1, 85) = 30.2, *p*<.001), HVLT-R acquisition (*F* (1, 85) = 13.5, *p*<.001), and HVLT-R delayed recall (*F* (1, 85) = 7.5, *p* = .007). When comparing the ADHD and TDC groups with FSIQ as covariate, the results from the ANOVA persisted. The main effects of diagnosis were as follows: verbal working memory (*F* (1,127) = 16.4, *p*<.001), HVLT-R acquisition (*F* (1,127) = 17.3, *p*<.001), and HVLT-R delayed recall (*F* (1,127) = 10.3, *p* = .002). A MANCOVA controlling for the overall effect of gender showed no significant effect of gender on the test results. Analyses excluding the participants on medication did not change the results.

### The results for the HFA+ group, the HFA- group, the ADHD group and the TDC group (see Table 4)

**Table 4:** Results on Letter Number Sequencing task (LNS) and Hopkins Verbal Learning Test—Revised (HVLT-R): means and standard deviations within the HFA+, HFA−, ADHD and TDC groups, and results from ANOVAs with post-hoc group comparisons reported.

Variable	HFA+^a)^	HFA-^b)^	ADHD	TDC	Group comparison			Post-hoc ^e)^
	(*n* = 16)	(*n* = 22)	(*n* = 79)	(*n* = 50)	*F*	*p*	η^2^	
LNS	13.4 (3.6)	16.0 (2.7)	15.5 (3.3)	18.2 (1.9)	*F*(3,163) = 14.3	*p*<.001	.21	HFA+ < ADHD, HFA-,< TDC
HVLT-Racquisition ^c)^	20.6 (7.7)	22.0 (5.6)	21.1 (5.9)	26.3 (4.7)	*F*(3,163) = 9.6	*p*<.001	.15	HFA+, ADHD, HFA- < TDC
HVLT-R recall ^d)^	6.6 (2.4)	8.1 (2.2)	7.5 (2.3)	9.1 (2.1)	*F*(3,163) = 7.5	*p*<.001	.12	HFA+, ADHD < TDC; HFA+ < HFA-

*Note*. HFA; High-functioning Autism: ADHD; Attention-Deficit/Hyperactivity Disorder: TDC; typically developing children: ^a)^ CBCL attention T-score>65: ^b)^ CBCL attention T-score≤65: ^c)^ sum trial 1, 2, 3: ^d)^ recall after 20 minutes. ^e)^ Fishers LSD.

ANOVAs revealed significant differences for all three test measures; verbal working memory: *F* (3,163) = 14.3, *p*<.001); HVLT-R acquisition: *F* (3,163) = 9.6, *p*<.001 and HVLT-R delayed recall: *F* (3,163) = 7.5, *p*<.001). Post-hoc analyses showed that the children with HFA+ were significantly (*p*<.05) more impaired than children with HFA-, ADHD and TDC on verbal working memory. In addition, children with ADHD and HFA- were significantly (*p*<.05) impaired compared to the TDC on verbal working memory. Both the children with HFA + and HFA - and the children with ADHD were significantly (*p*<.05) impaired compared to the TDC on HVLT-R acquisition. The children with HFA + and the children with ADHD were significantly impaired compared to the TDC on HVLT-R delayed recall (*p*<.001). Moreover, the children with HFA+ were also significantly (*p*<.05) more impaired than HFA- on this measure. ANOVAs excluding children on medication did not have an impact on any of the results. Independent measures T-test showed no significant differences between those in the ADHD group with a T-score below or above 65 on the CBCL attention subscale on any of the neurocognitive test measures.

## Discussion

Contrary to our expectations the children with HFA and the children with ADHD did not differ with regard to working memory. Nor did we find that the HFA group was more impaired than the children with ADHD on acquisition or delayed free recall. In fact, when comparing the undivided HFA sample with the ADHD group, the HFA and the ADHD groups performed quite similar. Both clinical groups were, however, impaired on all three neurocognitive test measures compared to the TDC group.

Regarding working memory, other studies have found that children with ADHD are more impaired than those with HFA on working memory [17], [19]–[20]. However, these studies differ from the current study in the working memory task used. Steele et al. [47] found that working memory deficits in HFA were load-dependent, and that only a complex task revealed difficulties. Our results may indicate that the LNS task provides the complexity needed to reveal possible difficulties in verbal working memory in children with HFA. This is coinciding with Leffard et al. [40] who state that the LNS task requires high processing demands. The results from our study on verbal working memory seems to be comparable with the results from Geurts et al. [21] who found no significant differences between children with ASD, ADHD and TDC on visual working memory. The children with HFA performed significantly impaired compared to TDC on measures of verbal working memory. This finding is in line with a review study by Russo et al. [48], finding deficits in verbal working memory in children with ASD compared to TDC.

On the acquisition and delayed recall tasks, the children with HFA displayed the same degree of impairment as did the children with ADHD on both measures, and not as expected less impairment on acquisition and more impairment on delayed recall. Acquisition as a main problem in learning has been found for the current ADHD group in a study by Andersen et al. [24]. The results from the current study indicate that the delayed recall deficits also in children with HFA may be secondary to deficits in acquisition, as was found in the children with ADHD [24]. If the delayed recall deficit was not secondary to the deficit in acquisition there should have been an additional impairment in delayed recall, reflected in a larger effect size that could account for both the impairment in acquisition plus the additional impairment in recall. Instead, finding that the impairment in delayed recall could be accounted for by the acquisition deficit, do not lend support to the hypothesis of Gras-Vincendon et al. [22]. They indicate that memory impairments in HFA are caused by recall problems instead of impaired acquisition. Our results are also contrary to the results of Phelan et al. [23] finding free delayed recall to be intact in children with HFA compared to TDC. However, as earlier stated, group comparisons between children with ADHD and children with HFA may be misleading since the HFA group is heterogeneous with regard to comorbid attention deficits.

Turning to the subdivision of the HFA group into those with and without attention deficit, we found that children in the HFA+ group were significantly more impaired compared to the HFA- group and the ADHD group on verbal working memory. This is in accordance with the results of Yerys et al. [49] finding high levels of ADHD-like symptoms in children with HFA to be associated with impairment in verbal working memory. Interestingly, the children with HFA+ were more impaired on verbal working memory than the children in the ADHD group. This may indicate an additive effect in children with combined HFA and attention problems causing significantly greater problems than in children with HFA without comorbid attention problems or in children with ADHD. Such an additive effect has also been found for inhibition [17]. Further comparisons between children with HFA with or without ADHD-like symptoms and children with ADHD are required in order to draw conclusions regarding neurocognition and comorbidity.

Although the children with HFA- performed better than children with HFA+ with regard to working memory, they were as impaired as the HFA+ and the ADHD group with regard to acquisition. The acquisition deficit (compared to TDC) of the HFA- group cannot be explained by symptoms of attention deficits in everyday life. Only the children with HFA+, and not the children with HFA-, were impaired compared to TDC on delayed recall. This may indicate that children with the combination of HFA and clinical attention problems have greater impairments than children with HFA without such symptoms, in verbal working memory and delayed recall. The results on delayed recall for the HFA- group support the findings of Phelan [23] that free delayed recall is intact in HFA.

Summing up, the inconsistent findings of neurocognitive deficits in children with HFA may be due to heterogeneity. More than half of the HFA sample performed normal on the CBCL attention rating. These children had significantly less impaired working memory score compared to those with HFA+ and no significant impairment compared to TDC on delayed recall.

It is important to note that the subgrouping of HFA children with or without attention deficit was not merely a way of dividing them into good functioning children on the one hand and more generally impaired children more similar to ASD. Although numerically lower IQ in the HFA+ group, there were no significant differences between the HFA subgroups with regard to IQ or total symptom load. It seems plausible that the subgrouping reflects a genuine heterogeneity limited specifically to symptoms of attention problems evident from the fact that even the HFA- group was impaired in working memory and acquisition. These neurocognitive impairments seems to be more a core deficit in children with HFA-, while the impaired delayed recall deficit in children with HFA+ is related to elevated attention problems.

### Clinical implications

Given that clinicians are routinely observing and treating individuals with co-occurring symptoms, treatment development will benefit from an enhanced understanding of learning and memory in children with pure HFA, children with HFA and clinically significant ADHD symptoms or children with pure ADHD [50]. Recent research shows that working memory can be trained using specifically designed computer programs [51], [52]. Whether children with HFA can profit from working memory training should be investigated in future research. Further, neurocognitive deficits may have a negative effect on both general functioning and academic achievement and make everyday life and school facilitation necessary.

Social skills training strategies are a widely used therapeutic approach in treating children with ASD, but such training has been found to have a limited effect on children and adolescents with ADHD [53]. It is possible that difficulties with working memory, acquisition and delayed recall along with different subtypes of ASD (HFA+ and HFA-) may complicate such interventions [53], [54] because it will take longer time to learn new social skills. Further, a successful social interaction often requires the ability to maintain focus and the ability to take-turns which may rely on intact working memory functions.

In conclusion, children with HFA whose parents report co-occurring ADHD symptoms in their children seem to differ from children with “pure” HFA with regard to learning and memory. This may support the need for a more dimensional way of looking at diagnoses, symptoms and everyday functioning in children with HFA, and have implications for treatment.

### Strengths and limitations of the study

Strengths of the present study are inclusion of groups of children with HFA, ADHD and TDC in the same study. Another strength is the division of the HFA group according to attention problems and the subsequent comparison between the clinical subgroups. The limitations are that the groups are not matched on gender ratio and the lack of measures for specific learning disabilities. Further, there are no children diagnosed with Autistic Disorder in the HFA group. Not applying ADI-R or ADOS criteria is a possible limitation of the study. Other potential limitations are that the sample was drawn from a clinical population, and represents those who are willing to seek help, and that we do not have information regarding those who refused to participate.
